# Phase II trial of preoperative radiochemotherapy with concurrent bevacizumab, capecitabine and oxaliplatin in patients with locally advanced rectal cancer

**DOI:** 10.1186/1748-717X-8-90

**Published:** 2013-04-15

**Authors:** Kathrin Dellas, Thomas Höhler, Thomas Reese, Florian Würschmidt, Erik Engel, Claus Rödel, Wolfgang Wagner, Michael Richter, Dirk Arnold, Jürgen Dunst

**Affiliations:** 1 Department of Radiooncology, University of Kiel, Kiel, Germany; 2 Department of Radiooncology, University of Luebeck, Luebeck, Germany; 3 Prosper Hospital Recklinghausen, Recklinghausen, Germany; 4 Martin Luther University Halle-Wittenberg, Department of Radiotherapy, Halle, Saale, Germany; 5 Private Practice of Radiooncology, Radiologische Allianz, Hamburg, Germany; 6 Private Practice of Hematology and Medical Oncology, Hamburg, Germany; 7 University of Frankfurt, Department of Radiooncology, Frankfurt, Germany; 8 Department of Radiooncology, Osnabrueck, Germany; 9 Coordination Center for Clinical Trials, Halle, Saale, Germany; 10 Clinic for Medical Oncology, Tumor Biology Center Freiburg, Freiburg, Germany

**Keywords:** Bevacizumab, Rectal cancer, Preoperative radiochemotherapy, Capecitabine, Oxaliplatin

## Abstract

**Background:**

Preoperative radiochemotherapy (RCT) with 5-FU or capecitabine is the standard of care for patients with locally advanced rectal cancer (LARC). Preoperative RCT achieves pathological complete response rates (pCR) of 10-15%. We conducted a single arm phase II study to investigate the feasibility and efficacy of addition of bevacizumab and oxaliplatin to preoperative standard RCT with capecitabine.

**Methods:**

Eligible patients had LARC (cT3-4; N0/1/2, M0/1) and were treated with preoperative RCT prior to planned surgery. Patients received conventionally fractionated radiotherapy (50.4 Gy in 1.8 Gy fractions) and simultaneous chemotherapy with capecitabine 825 mg/m^2^ bid (d1-14, d22-35) and oxaliplatin 50 mg/m^2^ (d1, d8, d22, d29). Bevacizumab 5 mg/kg was added on days 1, 15, and 29. The primary study objective was the pCR rate.

**Results:**

70 patients with LARC (cT3-4; N0/1, M0/1), ECOG < 2, were enrolled at 6 sites from 07/2008 through 02/2010 (median age 61 years [range 39–89], 68% male). At initial diagnosis, 84% of patients had clinical stage T3, 62% of patients had nodal involvement and 83% of patients were M0. Mean tumor distance from anal verge was 5.92 cm (± 3.68). 58 patients received the complete RCT (full dose RT and full dose of all chemotherapy). During preoperative treatment, grade 3 or 4 toxicities were experienced by 6 and 2 patients, respectively: grade 4 diarrhea and nausea in one patient (1.4%), respectively, grade 3 diarrhea in 2 patients (3%), grade 3 obstipation, anal abscess, anaphylactic reaction, leucopenia and neutropenia in one patient (1.4%), respectively. In total, 30 patients (46%) developed postoperative complications of any grade including one gastrointestinal perforation in one patient (2%), wound-healing problems in 7 patients (11%) and bleedings in 2 patients (3%). pCR was observed in 12/69 (17.4%) patients. Pathological downstaging (ypT < cT and ypN ≤ cN) was achieved in 31 of 69 patients (44.9%). All of the 66 operated patients had a R0 resection. 47 patients (68.1%) underwent sphincter preserving surgery.

**Conclusions:**

The addition of bevacizumab and oxaliplatin to RCT with capecitabine was well tolerated and did not increase perioperative morbidity or mortality. However, the pCR rate was not improved in comparison to other trials that used capecitabine or capecitabine/oxaliplatin in preoperative radiochemotherapy.

## Introduction

Preoperative radiochemotherapy (RCT) with 5-fluorouracil (5-FU) or capecitabine is the standard of care in many countries for patients with locally advanced rectal cancer (LARC) [1-4]. When followed by total mesorectal excision (TME), the risk of local relapse is 5-10% in patients treated with 5-fluorouracil (5-FU) and radiotherapy with 50.4 Gy. A pathological complete response (pCR) with these regimens is achieved in 10-15% of patients with acceptable toxicities. However, distant metastases occur in about a third of patients resulting in 10-year survival rates of 60% [5]. Therefore, there is a need to further improve treatment approaches to LARC.

A pCR after preoperative RCT is associated with favourable overall survival in rectal cancer patients and considered to be an appropriate early endpoint for evaluation of the effectiveness of intensified RCT-regimens [6-14]. In four randomized phase III trials oxaliplatin was added to 5-FU based preoperative RCT, but results will require further discussion [15-18]. The German CAO/ARO/AIO-04 trial which added oxaliplatin to 5-FU showed a small but significant improvement in pCR rate (17% vs. 13%). It has to be shown, whether these results further impact on decreased rates of local recurrences or distant metastases.

Bevacizumab (Avastin®; Genentech, Inc., South San Francisco, CA, USA), is a humanized monoclonal antibody against vascular endothelial growth factor A (VEGF-A), a critical and essential factor of angiogenesis, that promotes new vessel formations in tumors [19,20]. In metastatic colorectal cancer, chemotherapy combined with bevacizumab improves progression free and overall survival in 1^st^ and 2^nd^ line treatment. Preclinical data suggest that incorporating bevacizumab into preoperative RCT might improve the efficacy of radiotherapy [21].

Bevacizumab is associated with mechanism-based adverse events, for example, hypertension, gastrointestinal perforation, serious bleeding, thromboembolic events and wound-healing complications. Trials reported an increased risk of complications across all tumor types, which might be related to the VEGF blocking mechanism raising the question if the anti-VEGF-containing regimen may increase wound complications in the preoperative setting [22].

We initiated this prospective trial to evaluate the efficacy, safety and tolerability of adding bevacizumab to preoperative radiotherapy with a regimen of concurrent capecitabine and oxaliplatin (BevXelOx-RT) in patients with LARC. The pCR rate was the primary endpoint of this phase II study.

## Patients and methods

The study was conducted according to the principles of the Declaration of Helsinki and to good clinical practice guidelines. The Ethics Committee, University of Luebeck (No. 07–197) and all local review boards of the participating institutions approved this study. Each patient gave written informed consent before being accrued.

### Eligibility criteria

The eligibility criteria included histopathologically confirmed rectal cancer with the inferior margin within 16 cm from the anal verge, cT3-4 disease and/or positive perirectal lymph nodes without evidence of synchronous metastatic disease; however, a single resectable liver metastasis was not an exclusion criterion. Staging required endorectal ultrasonography and computed tomography of the pelvis, whereas the use of magnetic resonance imaging for staging was encouraged. Further inclusion criteria included Eastern Cooperative Oncology Group performance status <2, adequate renal, hepatic and hematologic function (creatinine clearance > 50 mL/min, total bilirubin concentration ≤ 2.0 mg/dL, liver transaminases and alkaline phosphatase concentration less than three times the upper normal limit and neutrophils > 2.5 x 10^9^/L). Patients were excluded if radiotherapy to the pelvic region or chemotherapy had previously been administered. Patients suffering from the following conditions were also ineligible: inflammatory bowel disease, malabsorption syndrome, history of other cancer, previous history of cardiac arrhythmia or coronary heart disease, peripheral neuropathy and psychiatric disorders or psychological disabilities thought to adversely affect treatment compliance. Pregnant or lactating patients and woman with childbearing potential who had lacked effective contraception were excluded.

### Pretreatment evaluation

Pretreatment evaluation included a complete history and physical examination, biopsy, digital examination, rigid rectoscopy, colonoscopy, endorectal ultrasound, computed tomography of the pelvis and abdomen and chest X-ray, ECG. Complete laboratory tests included a full blood count, blood electrolytes, creatinine, urea, liver transaminases, alkaline phosphatase, total bilirubin.

### Radiotherapy

Radiotherapy was delivered by a linear accelerator with a minimal energy of 6 MeV through a three- or four-field box technique to the primary tumor and mesorectal, presacral and regional lymph nodes up to the level of the fifth lumbal vertebra. The anal sphincter complex was included for low-lying tumors (< 6 cm from the anal verge). The dose was prescribed to a reference point according to ICRU 50 with the 95% reference-isodose covering the planning target volume. All patients received a total dose of 50.4 Gy, with daily fractions of 1.8 Gy on 5 days per week.

### Bevacizumab and preoperative RCT

**Figure 1:**
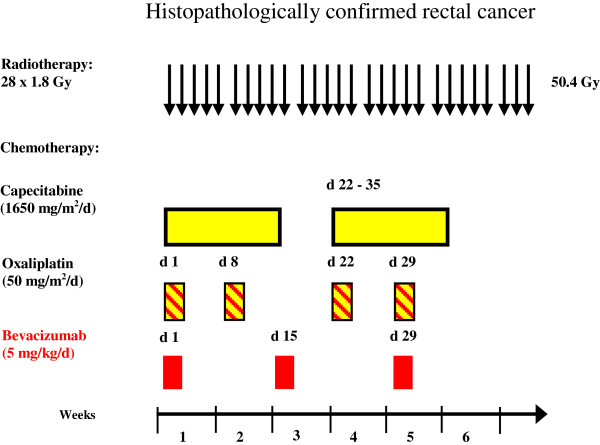
Overview of the study design and treatment schedule.

During preoperative therapy, bevacizumab was administered with 5 mg/kg body weight on days 1, 15 and 29. Capecitabine was administered at a fixed dose of 825 mg/m^2^ twice daily (30 min after breakfast and dinner) on days 1 to 14 and 22 to 35 of radiotherapy, and oxaliplatin as a 2-h infusion on days 1, 8, 22 and 29 at a dose of 50 mg/m, according to a schedule previously used in 2 phase II trials of our group [23-25] (Figure 1).

During treatment, patients were evaluated weekly regarding history, clinical examination, blood count, and biochemistry. Toxicities were assessed by National Cancer Institute Common Toxicity Criteria (NCI-CTC), version 3.0. We did not modify the radiotherapy schedule for grade ≤ 2 toxicities unless the severity worsened. Image-guided re-evaluation of the primary tumor was performed four weeks after the completion of preoperative treatment.

### Surgery and pathology

Four to six weeks after completion of radiochemotherapy with bevacizumab, TME was performed according to a standardized technique. If adjacent organs were involved intraoperatively, surgery was extended to partial or total resection of those adjacent pelvic organs. Central quality control of the surgery was not performed.

Pathologic complete response (pCR) was defined as the complete disappearance of viable tumor cells in the primary tumor and lymph nodes (ypT0N0). Histological regression was semiquantitatively determined according to a 5-point regression grading system established by Dworak by the local pathologist (no centralized analysis) [26].

### Adjuvant chemotherapy

Adjuvant chemotherapy was not mandatory by this protocol but it was recommended as monotherapy with capecitabine 1250 mg/m^2^ twice daily on days 1 to 14, repetition from day 22 for a total of 4 cycles.

### Study design, endpoints and statistical analysis

A prospective single-stage design according to Fleming was selected. The primary endpoint of the phase II study after preoperative BevXelOx-RT was pCR rate. Data of phase II trials with capecitabine, oxaliplatin and radiotherapy suggests pCR rates of 15-20%. We aimed to evaluate whether a 25% pCR rate could be achieved by adding bevacizumab to standard preoperative radiochemotherapy. A pCR rate of ≤ 15% was considered futile. With a sample size of 70 patients, the risk of erroneously claiming a major increase in activity despite a true pCR rate ≤ 15% (type I error) amounted to 10%, with a type II error probability of mistakenly rejecting BevXelOx-RT in the case of truly promising activity set of 20% corresponding to a power of 80% in a one-sided chi-square test. The secondary endpoints included radiographic response, pathologic downstaging, tumor regression grading, rates of sphincter-sparing surgery, toxicity (particularly postoperative surgical complications, risk of bowel perforation, wound healing complications and bleeding), rates of R0 resection and feasibility.

## Results

### Patient characteristics

A total of 70 patients were enrolled into the study at 6 investigation centers from July 2008 through February 2010. One patient was ineligible and excluded from the analysis because of serious uncontrolled physical or psychological disabilities. Therefore the intent-to-treat population is based on 69 patients. For 4 of these patients, the primary endpoint was not evaluated. The per-protocol-population consisted of 58 patients, who received the complete RCT. Safety analyses were performed within the 69 patients of the safety-population receiving at least on treatment application of the RCT. ITT- and safety population were not different from composition. The median age was 61 years (range 39–89), at initial diagnosis of the locally advanced rectal cancer 84% of patients showed clinical stage T3, 62% of patients had nodal involvement and 83% of patients were M0. The patient characteristics are listed in Table 1.

### Toxicity and dose modification

A total of 58 patients received preoperative BevXelOx-RT at the recommended dose level. Tables 2 and 3 show the frequencies and grades of the treatment-related toxicity.

**Table 1:** Baseline characteristics (n = 69)

**Characteristics**	**Value**
Age, years	
Median age	61.0
Range	39–89
Gender, n (%)	
Male	47 (68)
Female	22 (32)
ECOG performance status, n (%)	
0	47 (78)
1	13 (22)
TN clinical stage, n (%)	
T2N1-N2	2 (3)
T3N0	12 (17)
T3N1-N2	44 (64)
T4N0	3 (4)
T4N1-N2	4 (6)
M clinical stage, n (%)	
Mx	10 (14)
M0	57 (83)
M1	2 (3)
Tumor distance from anal verge, n (%)	
Mean ± SD (cm)	5.92 ± 3.68
Upper third (≥12 cm)	20 (33)
Middle third (6–12 cm)	26 (42)
Lower third (≤6 cm)	27 (43)

Abbreviation: ECOG = Eastern Cooperative Oncology Group.

Data presented as number of patients, with percentages in parentheses.

**Table 2:** Acute toxicities occurring during preoperative treatment

**Toxicities**	**Patients, n (%)**			
	**Grade 1**	**Grade 2**	**Grade 2**	**Grade 2**
**Hematological**				
Anemia	2(3)	1(1)	-	-
Leucopenia	7(10)	5(7)	1(1)	-
Thrombocytopenia	3(4)	1(1)	-	-
**Non-hematological**				
Diarrhea	17(25)	8(12)	2(3)	1(1)
Nausea	17(25)	5(7)	-	1(1)
Fatigue	15(22)	2(3)	-	-
Paresthesia	13(19)	2(3)	-	-
Obstipation	2(3)	-	1(1)	-
Anal abscess	-	-	1(1)	-
Anaphylactic reaction	-	-	1(1)	-
Hypertension	-	5(7)	-	-
Palmar-plantar erythrodysesthesia	8(12)	-	-	-
Pain	5(7)	2(3)	-	-

Adverse events were grouped whether they occured preoperatively and postoperatively. For both periods, 17 and 18 severe adverse events were reported, respectively. No perioperative death was documented.

The most frequent adverse event reported in the period up to one week after radiotherapy was in 31 patients (45%), of whom of 3 (4%) experienced grade 3 or 4 diarrhea, followed by nausea in 22 (32%) of patients, fatigue in 17 (32%), paresthesia in 15 (22%) and leucopenia in 13 (19%) patients. Any grade 3 or 4 toxicities were experienced by 6 and 2 patients, respectively: grade 4 toxicities included diarrhea and nausea and were restricted in one patient (1.4%), respectively. Grade 3 diarrhea occurred in 2 patients (3%) and grade 3 obstipation, anal abscess, anaphylactic reaction, leucopenia and neutropenia occurred in one patient (1.4%), respectively (Table 2).

**Table 3:** Postoperative acute toxicities

**Toxicities**	**Patients, n (%)**			
	**Grade 1**	**Grade 2**	**Grade 3**	**Grade 4**
**Hematological**				
Leucopenia	2(3)	1(1)	1(1)	-
**Non-hematological**				
Palmar-plantar erythrodysesthesia	14(20)	2(3)	1(1)	-
Fatigue	11(16)	2(3)	1(1)	-
Diarrhea	6(9)	2(3)	3(4)	-
Paresthesia	5(7)	5(7)	-	-
Ileus	-	-	-	1(1)
Nausea	2(3)	1(1)	1(1)	-
Proctitis	-	-	1(1)	-
Abdominal pain	-	-	1(1)	-
Anal fistula	-	-	1(1)	-
Pelvic abscess	-	-	1(1)	-
Sensory neuropathy	1(1)	4(6)	1(1)	-
Somnolence	-	-	1(1)	-
Delayed woundhealing	-	-	1(1)	-

The postoperative period was defined as the interval between surgery and the final examination four weeks after surgery. During this period, 43 (27%) adverse events occurred in 26 patients within the gastrointestinal tract system. The most common adverse event was the palmar-plantar erythrodysesthesia in 17 (25%) patients followed by fatigue in 14 (20%), diarrhea in 11 (16%) and paresthesia in 10 (14%) patients. The only grade 4 toxicity was ileus in one patient. Grade 3 toxicity occurred in 10 patients: diarrhea in 3 patients (4%) and nausea, proctitis, abdominal pain, anal fistula, pelvic abscess, sensoric neuropathy, somnolence, palmar-plantar erythrodysesthesia, fatigue, pain, delayed woundhealing and leucopenia in one patient (1.4%), respectively. Furthermore, we observed thrombosis in 3 patients (4%) and one deep vein thrombosis in one patient (1.4%) without stating grade (Table 3).

Among 69 patients commencing treatment, dose reduction or treatment discontinuations was necessary in 11 patients. 3 patients did not proceed to surgery (one patient with lost of follow up before surgery, one patient with progressive disease during preoperative treatment and exclusion from the study due to the protocol, one patient refused surgery), for one additional patient the pathological review was not assessable. For these 4 patients, the primary endpoint was not evaluated. In 9 patients, interruption of therapy was observed.

The mean relative dose intensity was high for bevacizumab (98.6%), oxaliplatin (97.8%) and radiotherapy (98.4%). For capecitabine being administered at a fixed dose of 825 mg/m^2^ twice daily on days 1 to 14 and 22 to 35 - according to the flat dosing scheme patients received 2000 mg/m^2^ to 3000 mg/m^2^. 62 of 69 patients (90%) received 100% of the recommended capecitabine, with dose reduction of capecitabine being observed in 7 patients.

### Efficacy

66 of 69 patients underwent surgery and achieved R0-resection (95.7%, Table 4). A pCR defined as ypT0N0 was observed in 12 of 69 patients for the intent-to-treat population (17.4%; 95% confidence interval, 10.4%-26.6%). Pathological downstaging (ypT < cT and ypN ≤ cN) was noted in 31 patients (44.9%, Table 5).

**Table 4:** Surgical procedures and resection status after preoperative BevXelOx-RT

**Characteristics**	**Value**
Type of surgery, n (%)	
Low anterior resection	28
Abdominoperineal exstirpation	19
Total mesorectal excision	13
Partial mesorectal excision	1
Mesorectal excision	1
Laparoscopic assisted rectal resection	1
Rectal exstirpation	1
Sigma-rectal exstirpation	1
Rectal resection with pouch	1
Resection status ^§^	
R0	66 (95.7)

Data presented as number of patients, with percentages in parentheses.

^§^ Refers to the primary tumor, 66 of 69 patients who underwent surgery.

Pathohistology information was available in 56 out of 66 patients (on treatment cohort). For 10 patients adequate data were not assessable. A complete regression of the tumor defined as ypT0 was documented in 11 patients (15.9% of the ITT cohort; 90% confidence interval, 9.2%-25.0%; 19.6% of the treatment cohort). 22 more patients (31.9% and 39.2% of the ITT cohort and on treatment cohort, respectively) showed good tumor regression (>50% of the tumor mass), moderate (n = 15, 21.7%), or minimal (n = 8, 11.6%) tumor regression, whereas no pathohistological response was observed in 3 patients (4.3%).

39 patients (56.6%) had no evidence for nodal involvement defined as pN0, 4 patients (5.8%) showed a good nodal regression, moderate (n = 2, 2.8%), minimal (n = 1, 1.4%) and no regression was noted in 4 patients (5.8%).

The sphincter preserving rate was 68.1% (47 of 69 patients). A temporary stoma was used in 13 patients (19.7%). In total, 25 patients (36.2%) developed postoperative complications of any grade including one gastrointestinal perforation in one patient (1.4%), wound-healing problems in 7 patients (9.8%) and bleedings in 2 patients (2.8%).

Follow up evaluation was limited to 6 months after initiation. Disease progression occurred in four patients. Two patients developed distant metastases (one lung, one liver) two months after initiation. For the third patient with progressive disease one month after initiation of therapy, adequate data regarding localization were not available. One patient with synchronous distant metastases (liver, lymph nodes) achieved tumor response (partial remission) with incomplete information of local lesion. 6 months after initiation all patients were alive.

## Discussion

We conducted this phase II trial as a multimodal regimen for patients with locally advanced rectal cancer, consisting of radiotherapy with concurrently administered chemotherapy with capecitabine, oxaliplatin and bevacizumab. This report summarizes the results of 69 patients and to the best of our knowledge, this is the largest trial in the multimodality therapy with bevacizumab-containing radiochemotherapy of rectal cancer patients in the preoperative setting.

**Table 5:** Postoperative pathological TNM stages compared with pretreatment clinical stages (n = 69)

	**Pathologic stage**										
**Baseline stage**	**ypTis**	**ypT0**	**ypT1**	**ypT2**	**ypT3**	**ypT4**	**ypTmissing**	**ypN0**	**ypN1**	**yPN2**	**ypNmissing**
T1 (n = 1)					1						
T2 (n = 3)				2	1						
T3 (n = 57)	1	12	3	14	24	2	1				
T4 (n = 5)				1	2	2					
N0 (n = 15)								13	1	1	
N1 (n = 41)								28	6	6	1
N2 (n = 8)								5	2	1	
Nx (n = 2)								1		1	

Regarding the primary endpoint of our phase II study, we failed to demonstrate a pCR rate considered to be “interesting”. The pCR rate of less than 18% was in the range of other reports investigating regimen with capecitabine alone or plus oxaliplatin in preoperative radiochemotherapy. Pathological downstaging (ypT < cT and ypN ≤ cN) was observed in 31 of 69 patients (44.9%).

Bevacizumab containing preoperative radiochemotherapy in rectal cancer has been investigated in a number of trials and appears to be safe and feasible with beneficial effects on vascular normalization, and promising response rates in smaller studies [27-33]. Velenik et al. [27] recently reported the efficacy of the addition of bevacizumab to capecitabine-RT in 61 patients with LARC. In this investigation, 13.3% achieved a pCR, and T-, N- and overall downstaging rates were 46.7%, 65.0% and 75.0%, respectively. Kennecke et al. [28] reported a similar pCR rate of 18.4% after a further triple treatment with bevacizumab, capecitabine, oxaliplatin and radiation. In 9 patients (23.7%) a complete regression of the tumor (ypT0) was documented including two patients with nodal metastases. Similarly, five of 25 patients (20%) had a pCR in the trial conducted by Dipetrillo et al. [29] using bevacizumab, oxaliplatin, 5-fluorouracil and radiation for rectal cancer. However, in this small study two cycles of induction mFOLFOX6 and bevacizumab were administered before concurrent bevacizumab, oxaliplatin, continuous infusion 5-fluoruracil and radiation.

Several phase II and III trials have shown that the combination of preoperative radiation with 5-FU/capecitabine and oxaliplatin shows moderately high rates of histopathological eradication of the tumor. Data of phase II trials suggested a pCR rate up to 20%, whereas results of phase III trials showed either no or only modest improvements with respect to the pCR rates [15-18,34,35]: the Italian STAR-01 study [16] as well as the US NSABP R-04 trial [17] and the French ACCORD study [18] did not show a significant increased rate of pCR´s, and in the German phase III trial, a small but statistically significant increment was documented [15].

The potential role of preoperative bevacizumab in combination with oxaliplatin remains still unclear. Preoperative bevacizumab could potentially impact the histopathological eradication alone or in combination with 5-FU or capecitabine since unprecedented synergistic or additive interaction between antiangiogenic and cytotoxic therapies initially has been reported in preclinical settings as well as in early phase I/II trials in terms of administration of bevacizumab alone or combined with 5-FU [21].

The addition of bevacizumab to radiochemotherapy with capecitabine and oxaliplatin did not lead to increased perioperative morbidity or mortality. The observed complications regarding quantity and kind of intervention after surgical treatment were within the expected range, without evidence of a modified spectrum of complications. In particular, we did not observe an increased number of bleeding complications, perioperative complications including anastomotic insufficiency or thromboembolic events. The most frequent adverse events reported in the period of BevXelOx-RT were chemotherapy-related, as diarrhea, nausea, fatigue, and paresthesia. In the postoperative period including surgery adverse events were mainly related to gastrointestinal tract system but also to pelvic abscess and delayed wound healing. No treatment related death was observed. With regard to perioperative complications in published bevacizumab-containing approaches in rectal cancer [29,30,32] we can conclude that bevacizumab does not increase the perioperative complication rate.

Potential benefits of bevacizumab may result in two different ways: to improve local control rate, which may be mirrored by the increased pathohistological response, and to prevent systemic metastases. However, as shown in this single arm phase II trial, the addition of bevacizumab to a complex multimodal regimen of a combination chemotherapy regimen and conventionally fractionated radiotherapy did not result in a clinically relevant increased pathohistologic response (namely pCR rate) compared to other reports. However, this somehow stands in contrast to the benefit of bevacizumab added to similar chemotherapy (FOLFOX or XELOX) regimens in metastatic colorectal cancer, when used as neoadjuvant therapy for liver metastases: Here, four phase II trials including more than 300 patients have reported higher pathohistologic response, including pCR rates [36-39]. Interestingly, the potential benefit of adding bevacizumab to standard chemotherapy was also seen in breast cancer, where even randomized trials reported increased pathologic complete response rates by more than 6% (34.5% vs. 28.2%) and overall clinical complete response rates by up to 8% (87.4% vs. 79.6%) [40,41].

It is not understood why this effect was not observed here; despite the preclinical and early clinical findings that bevacizumab may impact the pCR trials in terms of administration as monotherapy or in combination with fluoropyrimidines alone [21]. The potential benefit of adding bevacizumab to FOLFOX or XELOX in order to prevent patients with LARC from distant failure is questionable since bevacizumab in combination with oxaliplatin-based adjuvant therapy in patients with resected stage III or high-risk stage II colon carcinoma did not result in a clinical benefit [42,43].

Long term effects of this combined therapy regimen can not be judged by this trial and we also recognize the still ongoing discussion regarding the problem of using pCR as surrogate parameter in rectal cancer. Stage, tumor specimen, quality of pathologic analysis and the time period between therapy and surgery are considered to be key criterion for the impact on pCR. Several trials chosing pCR as a primary endpoint achieved lower rates of complete responses compared with historical data resulting from quality improvement of pathohistologic analysis [44]. The randomized phase II trial of Dewdney et al. demonstrated in KRAS/BRAF wild-type rectal cancer patients a significantly improved overall survival of the cetuximab arm without an increase of the pCR rate suggesting a possible benefit from systemic approaches before local therapy based on some not yet clearly understood biologic activity in this setting [44]. These effects regarding the long-term outcomes may also arise in the actual investigation.

However, at this time, long-term follow up results on survival and also on local control after preoperative radiochemotherapy with bevacizumab are needed in order to determine the potential impact of adding bevacizumab to preoperative standard treatment in patients with locally advanced rectal cancer. Furthermore, randomized trials are ongoing, in order to investigate the benefit of bevacizumab in addition to chemotherapy alone (without radiotherapy) in LARC.

## Conclusion

The results of our present clinical trial confirm that preoperative bevacizumab-based radiochemotherapy is feasible and well tolerated in LARC. However, the incorporation of bevacizumab into a combination regimen with 5-FU and oxaliplatin seems not to increase pathohistological response rates.

## Competing interests

This was an investigator-initiated trial supported in part by a grant from Hoffmann-La Roche GmbH, Darmstadt, Germany.

K. Dellas received speakers honoraria from Hoffmann-La Roche AG, Grenzach Whylen, Germany; C. Rödel received research funds from Hoffmann-La Roche AG, Grenzach Whylen, Germany and Merck Serono GmbH, Darmstadt, Germany and speakers honoraria from Merck Serono GmbH, Darmstadt, Germany; D. Arnold received research funds and speakers honoraria from Hoffmann-La Roche AG, Grenzach Whylen, Germany and Sanofi-Aventis Pharma GmbH, Berlin, Germany; J. Dunst received research funds and speakers honoraria from Hoffmann-La Roche AG, Grenzach Whylen, Germany.

## Authors’ contributions

KD, CR, MR, DA and JD contribution to design and supervision of the study. KD, TH, TR, FW, EE, CR, WW, DA and JD contribution to therapy and acquisition of data. MR contribution to acquisition of data and analysis. KD, CR, MR, DA and JD contribution to interpretation of data. KD drafted and reviewed the manuscript and all authors edited and approved the final version.
